# Mapping Sources of Assisted Dying Regulation in Belgium: A Scoping Review of the Literature

**DOI:** 10.1177/00302228231210146

**Published:** 2023-11-01

**Authors:** Madeleine Archer, Lindy Willmott, Kenneth Chambaere, Luc Deliens, Ben P White

**Affiliations:** 1 Australian Centre for Health Law Research, Faculty of Business and Law, 1969 Queensland University of Technology, Brisbane, QLD, Australia; 2 End-of-Life Care Research Group, 26656 Vrije Universiteit Brussel & Ghent University, Brussels, Belgium; 3 Department of Public Health & Primary Care, 26656 Ghent University, Ghent, Belgium

**Keywords:** assisted dying/suicide, euthanasia, Belgium, regulation, scoping review

## Abstract

Belgium has over 20 years of experience regulating assisted dying (AD). While much research considers this end-of-life practice, no studies have comprehensively analysed the various sources of regulation that govern it, including law, professional standards, and ethics. A scoping review identified all sources of regulation that guide AD practice, and their regulatory functions. Databases and reference lists were searched for records which met inclusion criteria between 11/2/22 and 25/3/22. Existing scholarship was used to identify sources of regulation, and thematically analyse their functions. Of the initial sample of 1364 records, 107 were included. Six sources of regulation were identified: law, policies, professional standards, training, advisory documents, and system design. Three regulatory functions were identified: prescribing conduct, scaffolding to support practice, and monitoring the system. The Belgian AD regulatory framework is multifaceted, complex, and fragmented. Providers must navigate and reconcile numerous sources of guidance providing this form of end-of-life care.

## Introduction

Assisted dying in some form is now legal in 13 countries. While most of these systems have been enacted in recent years, some jurisdictions now have significant experience regulating assisted dying (Cohen & Chambaere, 2022). Belgium is one such country with over 20 years of regulatory experience. The federal law creating a legal framework for assisted dying was passed in May 2002 and entered into force in September 2002.

The Belgian legislation (the Belgian Act) sets out several clinical and procedural conditions that must be satisfied before a physician can legally comply with a person’s written request for an assisted death (Table 1). Provided that each specific requirement is satisfied, the law allows adults with a serious and incurable medical condition causing constant and unbearable suffering to choose an assisted death. Minors who are assessed as having decision-making capacity to make this decision, and who satisfy certain additional conditions may also make this choice. The Belgian Act only refers to ‘voluntary euthanasia’, where a physician administers the lethal medication to the eligible person. It is silent on ‘physician assisted suicide’ or self-administered forms of assisted dying. An independent statutory body, the Federal Control and Evaluation Commission on Euthanasia (CFCEE) is tasked with reviewing each reported case of assisted dying after the person’s death, and reporting on that information to Parliament. While the Belgian Act is a federal instrument, implementation is the responsibility of both federal and regional levels of government. A unique feature of the legislation is that it expressly permits physicians to impose clinical or procedural conditions upon patient access in addition to those that the law prescribes (Vansweevelt, 2003).

**Table 1.** Summary of Legal Requirements in the Belgian Act.

	Requirements relating to the person and their condition	Requirements relating to the procedure
Adults^a^ who are clearly expected to die within the foreseeable future	• Legally competent • Conscious when making the request • Experiencing persistent and unbearable and hopeless physical or psychological suffering^b^ that results from a serious and incurable illness caused by accident or disease • Expected to die within the foreseeable future • Physician, together with the patient, believes that there are no reasonable alternative solutions for the person’s suffering, that the patient is suffering physically or mentally constantly and unbearably, and is certain of the durable nature of the person’s request, taking into account the progress of the person’s condition	‘Standard procedural requirements’ • The request for an assisted death must be voluntary, considered and repeated, and not arrived at as a result of any external pressure • Written request • Physician’s duty to give information (diagnoses and prognosis, all therapeutic options, the assessment procedure) • Consultation with an independent physician
Adults^a^ who are not clearly expected to die within the foreseeable future	• As above, but *not* clearly expected to die within the foreseeable future	• Standard procedural requirements (above) • Second consultation with an independent psychiatrist or specialist in the person’s condition • One month waiting period between the person’s written request and the provision of assisted dying
Minors whose death is foreseeable	• Has ‘capacity for discernment’ • In a medically futile state of constant and unbearable *physical* suffering that cannot be alleviated, and suffering is the result of a serious and incurable condition caused by illness or accident • Death is foreseeable • Conscious at the moment of making the request	• Standard procedural requirements (above) • Consultation with a child or adolescent psychiatrist or psychologist • Interview with the minor’s legal representatives, who give their written consent to the minor person’s request
Adults^a^ who have made an advance directive requesting an assisted death	• Legally competent at the time the advance request is made • Irreversible state of unconsciousness according to the current state of medical science • Serious and incurable condition caused by illness or accident and condition is irreversible given the current state of medical science	• Advance directive drafted and signed as prescribed • Consultation with an independent physician • Nursing team and adult confidant^c^ are consulted about the directive • The advance directive is valid for an indefinite period

^a^Under the Belgian Act, minors with legal capacity (or ‘emancipated minors’) are treated as adults. These are mature minors who, through marriage or a court decision have legal capacity to make their own decisions.

^b^‘Unbearable suffering’ is not defined in the Belgian Act. It is generally understood that whether the patient is experiencing unbearable suffering is based on the patient’s own subjective assessment, but the Belgian Act also prescribes that the physician must be believe that the patient is experiencing suffering that they consider unbearable.

^c^Under the Belgian Act, an adult confidant is a person who informs the person’s attending physician about the person’s advance directive requesting euthanasia.

In Belgium, individuals do not have a ‘right’ to an assisted death. A doctor can refuse to provide assisted dying, and no other person can be compelled to be involved in assisted dying. Institutions, for example, hospitals or nursing homes, can decide not to provide assisted dying services. However, an amendment to the Belgian Act dated 15 March 2020 prohibits institutions from preventing a doctor from providing assisted dying onsite.

The number of people receiving an assisted death in Belgium is increasing annually. In 2022, 2966 deaths were reported to the CFCEE, an increase of almost 10% from 2021. Most patients who accessed an assisted death were 70–89 years of age, suffered from cancer or several conditions, and their physician judged that their death was foreseeable. Requests for an assisted death from individuals whose sole or primary condition is psychiatric, requests based on an advance directive, and by minor patients are relatively few (Commission fédérale de Contrôle et d’Évaluation de l’Euthanasie, 2023).

Considerable research on the operation of the Belgian system has amassed in the past two decades. For example, this includes empirical evidence which demonstrates practical differences in the ways that assisted dying is implemented and practised in Belgium across different health care contexts. Different procedures and processes, which are not prescribed in the law, are employed by individuals and institutions when providing and assessing patients for an assisted death. For example, the Belgian Act does not specifically require patients to undergo palliative consultation and treatment prior to being assessed for, or eligible for, an assisted death. Despite this, evidence suggests that many healthcare institutions apply this ‘palliative filter’ (Gastmans et al., 2006). This means that patients who request an assisted death in some health care institutions must first consult with a palliative care team to discuss their request, and in some cases, only receive an assisted death if palliative options prove ineffective, insufficient, or if the patient strongly refuses undergoing palliative measures. Empirical evidence also reports that some of the procedural requirements set out in the law are not always complied with, including, consistently over time, the requirement to consult with an independent physician about the patient’s request (Cohen et al., 2014; Vissers et al., 2022). Differences in practice have also been found between the Flemish and Walloon regions; reporting rates, knowledge of, and adherence to the law differ between the regions (Cohen et al., 2012).

Limited research on the Belgian system is ‘regulatory,’ in the sense that it considers the sources through which regulation occurs and how these sources operate, including laws, policies and guidelines, ethical codes, training programs, professional standards, or funding programs (Vincent et al., 2020; White et al., 2022). Examining regulation is important. Regulation guides the behaviour of individuals and institutions and determines what specific behaviour is permitted, discouraged, mandated, or optional within a given regulatory environment (Braithwaite et al., 2007). In this way, regulation promotes consistent practice and compliance with the law.

Some scholarship specifically engages with regulatory sources operating in the Belgian system. Many scholars have investigated the Belgian Act itself, and some have investigated other laws which impact this legal framework (Delbeke, 2012). A significant body of work examines institutional ethics policies on assisted dying, including one important study which specifically considers the impact that these policies have on practice (Lemiengre et al., 2010; Verhofstadt et al., 2019a). Some scholars have considered the oversight and reporting roles exercised by the CFCEE, and the training and consultation roles played by Life End Information Forum (a regional, volunteer-initiated training and consultation service for physicians on end-of-life care) (Lewis & Black, 2013; Van Wesemael et al., 2009). However, work to date has examined only a small subset of regulatory sources, and it has tended to examine a specific source of regulation in isolation from others. What is missing, therefore, is comprehensive examination of all sources of regulatory influence, and a wider understanding of the role regulation plays within this system.

Designing effective end-of-life care and evaluating existing regulation necessitates viewing regulation holistically. Existing literature has tended to take a piecemeal, or siloed approach to examining regulation, by considering just law, or just policy, or just training. This approach can lead to sub-optimal outcomes for patients, families, health professionals, and health systems, as it does not recognise that regulatory sources interact with one another to guide behaviour (White et al., 2022).

Understanding what regulatory sources comprise the Belgian assisted dying regulatory landscape facilitates a holistic understanding of the forces which guide doctors, nurses, pharmacists, other caregivers, institutions, patients, and their families to make decisions about assisted dying. Consolidating regulatory learnings may explain why processes differ institution to institution, and provider to provider. It might also illuminate why regulation does not guide behaviour in all situations, or why sources of regulation may fail to guide behaviour as intended. Finally, it may facilitate cross-cultural comparisons with assisted dying regulatory landscapes in other jurisdictions, including where assisted dying regulation is newer (Close et al., 2021). Namely, these learnings may enable policymakers internationally to identify the sources of regulation which shape assisted dying practice in their jurisdiction, their provenance, and the functions that each source plays in regulating assisted dying. This in turn might assist policymakers to consider the impact that regulation is having on assisted dying practice.

In this article, we systematically scoped the scholarly literature on the Belgian assisted dying regulatory framework. Our aim was to map the sources of regulation which seek to impact assisted dying practice in Belgium and to identify their regulatory functions. This approach leverages existing work which considers regulatory sources separately, and integrates learnings about the provenance and nature of these sources. This scoping review sought to answer two research questions. First, what are the sources of regulation operating in the current Belgian assisted dying system including, but not limited to law, policy, ethical codes, and professional standards? Second, what are the regulatory functions that these sources perform?

## Methods

We used scoping review methodology to systematically and comprehensively map the literature on the Belgian assisted dying system through the lens of regulation. Scoping reviews are useful tools for summarising the current state of understanding on a particular topic, identifying what is known on a particular topic, and answering exploratory research questions (Anderson et al., 2008). We collaboratively developed a study protocol setting out the initial aims and methods for the review and iteratively adapted our approach in response to findings from progressive analysis (see Supplemental material 1).

The review was guided by Arksey and O’Malley’s methodological framework for scoping studies (Arksey & O’Malley, 2005). Accordingly, we identified the research questions; identified, and selected relevant studies; charted the data; and collated, summarised, and reported the results. Where the methods undertaken permitted, this review is reported consistent with the PRISMA-ScR guidelines (Tricco et al., 2018).

### Defining Regulation, Regulatory Sources, and Regulatory Instruments

We adopted Black’s widely used and authoritative definition of regulation to answer the research questions and identify relevant regulatory sources: ‘regulation is the sustained and focused attempt to alter the behaviour of others according to defined standards or purposes with the intention of producing a broadly identified outcome or outcomes, which may involve mechanisms of standard-setting, information-gathering and behaviour-modification’ (Black, 2002). This definition envisages that a broad range of sources might regulate practice, and existing scholarship in healthcare regulation recognises this might include laws, policies and guidelines, ethical codes, training programs, funding programs, and professional standards (Vincent et al., 2020; White et al., 2022). This definition of regulation would exclude, however, other more intangible factors such as culture where they lack the intent to guide behaviour (even if they might do so). We also note the three functions that regulatory sources might have, namely, setting standards, gathering information, and modifying behaviour and the nature of these are discussed below.

Both instruments (e.g. the law) and organisations (e.g. the CFCEE) were considered regulatory sources where they satisfied the above definition of regulation. However, there was a focus on regulatory instruments because organisations’ regulatory activities are expected to be making and disseminating instruments such as policies and guidelines. Further, while we recognise the critical role that individuals play within regulating organisations, individuals as regulators were excluded as this study aimed to map structures and systems. Finally, international forms of regulation (such as international conventions) were excluded from this study due to their tangential relevance to Belgian assisted dying practice.

### Identifying Relevant Studies

#### Database Searching

Between 11/02/22 and 25/03/22 we searched six databases, Scopus, PubMed, Medline, Cinahl, PsychInfo, and Legal Source. These databases were selected because they index research in law, medicine, health, psychology, and social science. We framed the search strategy in broad terms to capture a large sample of potentially relevant records. We applied the search strategy to titles and abstracts. Records were included if they contained two essential components: references to both assisted dying and Belgium (and variations in terminology). Where possible in each database, we applied searching filters to reflect the formal inclusion criteria (discussed below). For illustration, the search strategy applied in PubMed was:

(euthanasia[Title/Abstract] OR “voluntary euthanasia”[Title/Abstract] OR “assisted dying”[Title/Abstract] OR “assisted suicide”[Title/Abstract] OR “assisted death”[Title/Abstract] OR “physician assisted suicide”[Title/Abstract] OR “physician assisted death”[Title/Abstract] OR “physician assisted dying”[Title/Abstract] OR “mercy killing”[Title/Abstract] OR “medical assistance in dying”[Title/Abstract] OR “medical aid in dying”[Title/Abstract]) AND (belg*[Title/Abstract] OR flemish[Title/Abstract] OR flanders[Title/Abstract] OR walloon[Title/Abstract] OR wallonia[Title/Abstract] OR benelux[Title/Abstract])

The year of publication was restricted to 2002–2022 and journal articles were sought in English, Dutch, and French. Two further search strategies were applied in the same databases. The first substituted French and Dutch terms for the English equivalents (Table 2), and the second extended the search to book chapters in English. The full search strategies and the dates on which each search was undertaken are contained in Supplemental material 2.

**Table 2.** English, French, and Dutch database Search Terms.

Search term	English	French	Dutch
Assisted dying	euthanasia or voluntary euthanasia or assisted dying or assisted suicide or assisted death or physician assisted death or physician assisted dying or mercy killing or medical assistance in dying or medical aid in dying	euthanasie or aide medicale a mourir or aide au suicide or aider a mourir	euthanasie
Belgium	belg* or Flemish or Flanders or Walloon or Wallonia or benelux	belg* or flamand or flandre or wallonie or benelux	belg* or vlaam or vlaanderen or waals or wallonie or benelux

We imported records generated by the database search strategy into Zotero and removed duplicates. We then applied an eligibility review process to all records, comprising title, abstract, and full-text review (discussed below).

#### Reference List Searching

Reference lists and footnotes were an additional data source. The reference lists and footnotes of records which were included after full text review were scanned for relevant records not already identified. Citations were subject to the standard eligibility review process if they met the formal inclusion criteria, and explicitly concerned both assisted dying and Belgium.

### Inclusion and Exclusion Criteria

Both formal and substantive inclusion criteria were applied to the records (Table 3). Records were required to be published after the Belgian Act became law, to exclude assisted dying practices or regulation prior to the enactment of the legal framework. Records were required to be published in English, Dutch, or French. English was an appropriate language to include given the intentional dissemination of research by Belgian researchers in English languages outputs. This approach to language is consistent with similar Belgian reviews in the end-of-life field (Andrew et al., 2013). In relation to the substantive inclusion criteria, ‘assisted dying’ referred to all practices legalised pursuant to the Belgian Act. As such, forms of assisted death outside of the legal framework, such as palliative sedation, and unlawful killings, were not captured.

**Table 3.** Formal and Substantive Inclusion Criteria Applied in the Review.

Formal Inclusion Criteria	Book chapter or journal article in a peer reviewed^a^ journal
	Full text available
	Published 28 May 2002 (inclusive) to 2022 (the date on which the relevant search was conducted)^b^
	Written in English, Dutch, or French
Substantive Inclusion Criteria	Belgian regulation of assisted dying is a substantive focus of the record
	Record has substantive discussion, analysis, or engagement with one or more specific regulatory sources operating in the present Belgian assisted dying legal framework

^a^A journal was considered to be peer reviewed unless web searching suggested that it was not. Ulrichsweb Global Serials Directory was used to facilitate this process but the absence of a ‘refereed’ icon was non-determinative of a lack of peer review and further searching was undertaken.

^b^The end-dates for each of the three searches were 11/02/22, 09/03/22 and 25/03/22.

### Study Selection

All records were subject to a title, abstract, and full-text review process throughout which they were assessed against the inclusion criteria. A research journal was used to document decision-making.

An inclusive approach was taken at the title review phase. Only clearly irrelevant records, or records which did not meet the formal inclusion criteria were excluded.

Abstract screening was a more robust review phase. Both the formal and substantive inclusion criteria were applied to the abstract for each record. The abstract had to itself satisfy both substantive inclusion criteria, or strongly indicate that the full-text would satisfy both substantive inclusion criteria in order to progress to full-text review. Records proceeded to full-text review where they did not have an abstract, or where it was not accessible. MA and BPW co-assessed a random sample of 30 abstracts. This process was undertaken blind to the other reviewer’s assessment as to eligibility. Disagreements were resolved by discussion and reaching a joint conclusion. MA then undertook the remainder of the abstract screening process.

The substantive inclusion criteria were then applied to the full text of records remaining in the sample. MA and BPW moderated a sample of 10 records using the above moderation procedure. Records which satisfied all the formal and substantive inclusion criteria after the full-text review phase constituted the ‘scoping review sample’ and were subject to data extraction and analysis.

### Data Extraction

MA (health law and regulation scholar) undertook the data extraction and analysis phases in consultation with BPW (health law and regulation scholar) and LW (health law and regulation scholar). The first step was data familiarisation. All records were read before descriptive information about the record was entered into a Microsoft Excel spreadsheet (Descriptive Data Extraction Tool). The descriptive information extracted for each record included the record’s citation, study type, language, focus, and the regulatory sources it referred to (collected after data analysis took place).

The translation software ‘DeepL’ was used to translate the French and Dutch records into English so that they could be read and analysed. All translations were of sufficient quality to identify the regulatory sources referred to and the nature of the discussion about them.

Records were read a second time, and potential regulatory sources, for example, laws, policies, and guidelines, were identified and highlighted. This identification process was guided by scholarship highlighting the regulatory sources likely to operate in this system (Vincent et al., 2020; White et al., 2022). Each potential regulatory source was then assessed against the definition of ‘regulation’ used in this study. Provided this test was satisfied, the regulatory source, and information about it were extracted into a second Microsoft Excel spreadsheet (Regulatory Data Extraction Tool). Sufficient information had to be provided in the text of the record to discern whether the source met the study’s definition of regulation, and where detail was insufficient, the source was not included.

Data extraction was an iterative process: the repetitive nature of the data meant that it was possible to progressively extract and synthesise existing learnings and to avoid duplication in the Regulatory Data Extraction Tool. For example, the Belgian Act was only entered once into the Tool, and only new information about it provided by subsequent records was added as data was progressively extracted from each record. The record which mentioned each regulatory source was also recorded in the Tool.

### Collating, Summarising, and Reporting the Results

The data extraction and analysis processes overlapped. This is because the Regulatory Data Extraction Tool functioned as a template or coding frame which facilitated data analysis.

To address the study’s first research question, a coding frame was applied to all records to hierarchically map the identified regulatory sources. This approach drew on template analysis, a codebook approach to thematic analysis in which a template is developed as a tool to produce a hierarchical thematic structure (King, 2004). This mapping process was informed by several a priori codes identified deductively from the literature, consisting of law, policy, guidelines, ethical codes, training programs, professional standards, and funding programs (Vincent et al., 2020; White et al., 2022). As a new regulatory source was added to the coding frame, it was sorted into these categories. These categories were refined, hierarchically organised, or removed throughout the analysis process. For example, the Belgian Act was sorted into the ‘law’ category, and as case law and royal decrees emerged, ‘legislation’, ‘royal decrees’ and ‘case law’ sub-categories were formed.

To answer the study’s second research question, information about the regulatory functions of each regulatory source was also extracted into the Regulatory Data Extraction Tool. This data was also thematically analysed within the coding frame using template analysis to generate semantic themes. First, the data was coded into the functions played by each regulatory source. For example, ‘adjudicating disputes’, ‘process-setting’, and ‘gathering data.’ These codes were then combined, refined, and reorganised considering Black’s three conceptions of the functions of regulation which functioned as a priori themes: standard-setting, information-gathering, and behaviour modification (Black, 2002). This analytical process was therefore both deductive and inductive, and themes were iteratively adapted as the data was analysed in order to reflect the regulatory functions performed by each regulatory source. For example, the ‘information-gathering’ a priori theme was subsumed into a broader ‘monitoring the system’ theme as the data was analysed.

After data extraction and analysis were complete, we analysed the Descriptive Data Extraction Tool to produce descriptive statistics for the review sample.

## Results

**Figure 1.**
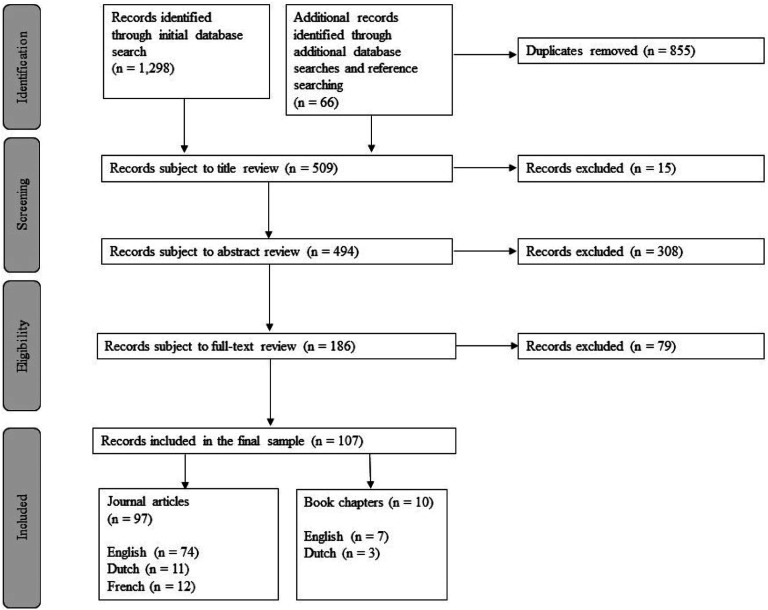
Flowchart of the outcomes of the eligibility review process.

Of the initial 1364 records generated by the database and reference list searches, 107 records were included (Figure 1). Records were excluded at the abstract and full-text review phases if they did not meet the formal or substantive inclusion criteria. For example, the focus of the record was attitudinal, not regulatory, or its focus was on reporting statistics, not investigating regulation.

Most records included in the review were journal articles (91%), and most were written in English (76%) (Table 4). Most of the included records were non-empirical works (69%), meaning they did not undertake empirical research and were doctrinal/legal or comparative analyses, brief articles, ‘forum’ or ‘news’ articles, or letters to the editor. Of the 74 non-empirical works, four of these included a regulatory tool which met the definition of a regulatory source in this study, for example, an institutional policy was extracted at the end of the journal article. The descriptive information extracted from each record is contained in Supplemental material 3.

**Table 4.** Review Sample Descriptive Statistics.

Sample characteristics		N = 107 (%)
Record type	Journal article	97 (91%)
	Book chapter	10 (9%)
Language	English	81 (76%)
	Dutch	14 (13%)
	French	12 (11%)
Study type	Empirical	33 (31%)
	Non-empirical	74 (69%)

We identified six overarching regulatory sources which operate in the Belgian assisted dying system, as guided by relevant regulatory scholarship (Vincent et al., 2020; White et al., 2022). The regulatory sources identified are law, policy, professional standards, training programs, advisory documents, and system design. Sub-categories for each of these overarching sources, and their provenance, were also identified (see Supplemental material 4).

Law was the most discussed regulatory source. Every record cited the law’s role in regulating assisted dying in Belgium (Table 5). The next most discussed regulatory source was system design (67%), followed by professional standards (40%), training programs (35%), policy (27%), and advisory documents (7%). It was uncommon that records discussed more than four regulatory sources (Table 5). Most records discussed three regulatory sources (38%), followed by two (28%), one (14%), four (12%), five (6%), and six (2%).

**Table 5.** The Extent to Which Regulatory Sources Were Discussed in the Review Sample.

Regulatory source(s) discussed per record	Number of records (N = 107) (%)
Law	107 (100%)
System design	72 (67%)
Professional standards	43 (40%)
Training programs	37 (35%)
Policy	29 (27%)
Advisory documents	7 (7%)
Number of regulatory sources discussed per record	
1	15 (14%)
2	30 (28%)
3	41 (38%)
4	13 (12%)
5	6 (6%)
6	2 (2%)

We also generated three themes which describe the regulatory function of the identified regulatory sources. These are prescribing conduct, scaffolding the system, and monitoring the system. These themes were generated by adapting Black’s three conceptions of the functions of regulation to the data being analysed (Black, 2002).

### Sources of Regulation

#### Law

Law was the most prominent regulatory source discussed in the review sample. This overarching regulatory source has three sub-categories: legislation, case law, and royal decrees. Legislation is made by Parliament and consists of the Belgian Act and three amendments to the law. The 2005 amendment clarified the role and obligations of pharmacists who dispense and deliver medication used for assisted dying. The 2014 amendment extended the law to minors with the capacity to make decisions about assisted dying. The 2020 amendment concerned conscientious objection and referral obligations, institutional objection, and the validity of advance directives on assisted dying.

In the case law sub-category, cases of the constitutional court in 2004 and 2015 considered the constitutional validity of the Belgian Act and the extent to which it adheres to human rights principles. In the criminal jurisdiction, two cases in Ghent in 2004 and 2020, and one case in Liège in 2003 were described, which alleged misconduct of a health professional pursuant to the assisted dying legal framework.

The Crown issues royal decrees, which confer legislative power on the executive. The decrees discussed in the sample concern the form, format, and accessibility of advance directives on assisted dying, and the appointment of members of the CFCEE.

#### Policy

This regulatory source is comprised of three sub-categories: organisational policies, institutional policies, and public policy. Organisational policies are produced by health care umbrella organisations and networks. These organisations are Caritas Flanders, Zorgnet-Icuro, Palliative Care Flanders (formerly the Flemish Federation for Palliative Care), the Fédération Wallonne des Soins Palliatifs, the Fédération Pluraliste Bruxelloise de Soins Palliatifs et Continus, and the Brothers of Charity.

Institutional policies are produced by hospitals and residential aged care facilities. They were referred to in the review sample both collectively and individually. For example, one study investigated ‘ethics policies on euthanasia in Flemish hospitals’ and another the ‘Ghent University hospital procedure concerning euthanasia and psychological suffering’ (Lemiengre et al., 2007; Verhofstadt et al., 2019b).

The public policy sub-category describes a circular for doctors entitled ‘advance requests for euthanasia – electronic consultation’ published in 2008 by the Federal Health Department.

#### Professional Standards

This regulatory source has two sub-categories: written professional guidelines and disciplinary proceedings. The written guidelines are position statements, specific written opinions, or deontological codes which refer to assisted dying, concerning the medical, psychiatric, general practice, or pharmaceutical professions. They are produced by the Belgian National Council of Physicians, the Flemish Association for Psychiatry, the Walloon Royal Society of Psychiatric Medicine of Belgium, the Scientific Society of Flemish General Practitioners, and the Association Pharmaceutique Belge.

One 2007 disciplinary proceeding was referred to in the review sample. This matter was initiated by the Provincial Council of West Flanders and investigated whether a health professional’s act was an assisted death and therefore regulated by the legal framework.

#### Training Programs

The training overarching regulatory source has two sub-categories: mandatory and non-mandatory training programs. The mandatory training referred to in the review sample was the assisted dying component of the Free University Brussels’ (VUB) core medical undergraduate curriculum. Non-mandatory training consists of the VUB’s master’s certificate in pain at the end-of-life, a post-graduate course run by Palliative Care Flanders, and two regional training programs specific to end-of-life care and assisted dying. Life End Information Forum (LEIF) and Forum End of Life (Forum EOL) operate in Flanders and Wallonia respectively. They are community-initiated programs which provide education and support for physicians on end-of-life care and assisted dying. Both LEIF and Forum EOL also engage in other regulatory activities, discussed below.

#### Advisory Documents

This regulatory source comprises non-legally binding clarifications or interpretations about specific issues in practice. This source has two sub-categories: documents concerning assisted dying practice authored by independent statutory bodies, and academic writings. The independent statutory bodies who have written on assisted dying, namely, the CFCEE and the Belgian Advisory Committee on Bioethics (BACB), have different mandates. The CFCEE drafts biannual reports on assisted dying practice which it remits to Parliament and which are widely disseminated, and it also produces an information brochure for physicians. The BACB is an advisory body which generates and disseminates opinions on problems in biology, medicine, and health care identified in research. Its investigation can be self-initiated, or requested by a government or other organisation (Belgian Advisory Committee on Bioethics, 2019). Two of the BACB’s opinions were discussed in the sample. They relate to the legal validity of institutional objection to assisted dying, and the permissibility of assisted dying for patients with mental illness.

The academic writings sub-category describes a 2006 academic paper written by a GP, widely disseminated to doctors and which provides specific information on the technical provision of assisted dying.

#### System Design

The system design overarching regulatory source describes sources and infrastructure which perform a structural role in the regulatory framework. These sources support the translation of the Belgian Act into practice and make the system workable. Some of this infrastructure is initiated by government, and some is not. There are three sub-categories: system design which pre-dates the Belgian Act and is pre-existing systemic infrastructure, system design created by the legal framework for assisted dying, and system design for assisted dying which was developed independently of the Belgian Act.

Pre-existing system infrastructure refers to the public prosecutor and its role in assisted dying. This office was established before, and independently of, the Belgian Act but it receives and investigates referrals from the CFCEE where non-adherence to the law is suspected. It can also self-initiate investigations.

System design created by the Belgian Act consists of the CFCEE and its internal mechanisms (including the CFCEE secretariat and the CFCEE registration form). A second source of regulation created by the legal framework is a national database for the registration of advance requests for assisted dying.

In terms of system design which exists independently of the Belgian Act, LEIF and Forum EOL operate consultation services which facilitate doctors who receive a request for an assisted death to consult an expert or connect with an independent physician who may undertake the legislatively prescribed consultation. These services also provide doctors with information on assisted dying and other medical end-of-life decisions. The review sample also referred to ‘consultation centres’ which provide information to patients and health professionals about assisted dying and give patients another avenue for seeking assistance to die. These centres are a contingency plan for some individuals, as they receive and handle requests from individuals who cannot address their requests to their attending physician. For example, Ulteam is an end-of-life service for patients, especially those with mental illness, who have been refused access to assisted dying by their doctor. This service acts as a form of ‘second line’ consultation, and patients can be admitted there (Behaegel et al., 2015).

### Functions of the Identified Regulatory Sources

Three themes were generated in relation to the functions played by the identified regulatory sources: prescribing conduct (prospectively and retrospectively), scaffolding the system, and monitoring the system.

#### Prescribing Conduct

This theme refers to setting standards for practice, prescribing, or defining roles, responsibilities, rights, obligations, expectations, processes, or necessary skills in relation to assisted dying. All regulatory sources identified in this study have this function.

The term ‘prescribing’ is used irrespective of the degree and nature of prescriptiveness of the command. For example, laws and policies both prescribe conduct, though laws are legally binding, and policies are not. Similarly, some commands are clinical, legal, ethical, or relating to process, but they all prescribe conduct that should occur.

Law creates a medico-legal framework for assisted dying in Belgium. It defines the conditions under which conduct is protected (thereby defining illegal or unregulated conduct), distinguishes assisted dying from other end of life acts, and establishes procedural and eligibility conditions for legal access. It also sets out rights and responsibilities, defines how relationships should be managed, puts in place mechanisms for implementation and the ongoing management of the framework, and establishes assisted dying as a clinical practice in the medical sphere.

Policies, professional standards, training programs, and advisory documents all prescribe conduct by engaging with the law and interpreting it, operationalising it, and providing practical advice on navigating the legal framework. They reinforce and replicate the prescriptions set out within the Belgian Act and prescribe their own eligibility and procedural conditions by adding qualifications or stipulating the proper process at various stages. By rendering a step-by-step guideline for employees, institutional policies are particularly prescriptive as to the correct procedure that physicians and other employees must undertake to retain the protection of the law and prevent illegal conduct.

Professional standards are also concerned with setting and enforcing standards with a view to protecting health professionals who provide assisted dying. Policies delineate caregiver roles, support health care workers to make choices about their participation, and emphasise health professionals’ roles and responsibilities. Professional standards enumerate health professionals’ rights, duties, and obligations. Relatedly, policies ensure that health professionals’ conduct is in line with institutional regulations, and professional standards ensure consistency with clinical and professional norms.

The conduct that policies and professional standards prescribe are inherently tied to their stances on assisted dying and the legal framework. Their stance determines which patients might access an assisted death, and what procedures are in place for each patient’s assisted dying assessment trajectory. The conduct that professional standards and training programs prescribe are also linked to professional best-practice approaches in clinical care and administration methods.

Policies, professional standards, and training programs also prescribe conduct relating to care, carefulness, and the patient experience. Some policies prescribe processes and conduct for patients who are found ineligible, aftercare, and patient and relative involvement in the assisted dying decision-making processes. They emphasise that care must be high-quality, consistent, and ethical. Training programs encourage communication between the patient and their care team and within the patient’s care team.

Advisory documents prescribe conduct by providing important clarification on assisted dying practices. By reporting the conditions from which eligible patients have suffered, and legitimate steps taken in relation to procedure and administration, the CFCEE reports set important standards for physicians. Similarly, by adjudicating on the legality of, for example, institutional policies which do not provide assisted dying services, the BABC might similarly alter physicians’ and institutions’ conduct in the provision of assisted dying, though in a less direct sense than, for example, the Belgian Act.

In terms of system design, the CFCEE registration document sets the standard for what specific information is reported to the CFCEE in respect of each death. The existence of the database for advance directives informs physicians to archive and access these documents.

Some regulatory sources prescribe conduct retrospectively. This includes modifying behaviour by applying consequences to conduct which has already occurred, for example, by rewarding appropriate behaviour or sanctioning conduct that is inappropriate or illegal. Case law retrospectively prescribes conduct by adjudicating legal disputes and applying and enforcing the Belgian Act. In the criminal law sphere, judgements determine which conduct is legal and which is illegal, and infringing individuals may receive punitive sanctions. In the same vein, disciplinary proceedings in the professional sphere apply medical and ethical deontology to factual situations. In this way, professional bodies determine which conduct is professional or unprofessional, and might apply appropriate disciplinary sanctions.

System design, in terms of the public prosecutor and the CFCEE also retrospectively prescribes conduct. The public prosecutor enforces the provisions of the Belgian Act by conducting investigations and prosecuting potential offenders. The CFCEE does not itself enforce the Belgian Act*,* but by acting as a buffer between the medical and criminal spheres, facilitates the public prosecutor’s role and in this way indirectly sanctions physicians’ conduct.

#### Scaffolding the System

This theme refers to sources and infrastructure with the function of translating the law into practice, making the system workable, practical, and accessible. Amendments to the law and royal decrees scaffold the system by ensuring pharmacological supply, establishing referral obligations, clarifying processes for the registration of advance directives on assisted dying, and establishing processes for appointing members of the CFCEE. The identified public policy supports the requirement to register advance directives on assisted dying by providing information and processes for doing so. Written professional standards for pharmacists also ensure the availability of medications for assisted dying and pharmacists willing to dispense the medication. Training programs teach physicians how to navigate and comply with the legal framework. All forms of system design, by their nature, scaffold the assisted dying system, though some mechanisms are legally ordained and ‘top down’, and others are unofficial or ‘bottom up’ scaffolds. Consultation centres are an example of the latter. By facilitating the independent consulting assessment required by the law, they scaffold the law’s implementation and exist to make the system workable.

#### Monitoring the System

This theme refers to the regulatory function of monitoring the operation of the assisted dying system by gathering information about it, reporting on, and disseminating this information, and using it to exert social control over this practice.

The only regulatory sources identified in this study which engage in information gathering are system design (the CFCEE) and advisory documents (the CFCEE’s biannual reports). The CFCEE monitors and evaluates each reported case of assisted death, compiles this information, and reports this data to Parliament and the wider public, to evaluate assisted dying practice and identify trends. They use this collated information to make recommendations to Parliament regarding assisted dying practice.

## Discussion

### Main Findings of the Review

This study identified the sources of regulation which seek to guide behaviour within the Belgian assisted dying system. The six regulatory sources identified in this regulatory framework are law, policy, professional standards, training programs, advisory documents, and system design. Some of these forms of regulation are well-known and well-studied, including the Belgian Act*,* LEIF, and the CFCEE. Other regulatory sources are less well-studied, including amendments to the law, court decisions, royal decrees, public policy, and advisory documents.

This study employed thematic analysis to identify the functions that these identified regulatory sources have within this system. These functions are prescribing conduct (prospectively and retrospectively), scaffolding the system, and monitoring the system. All six regulatory sources operating in this system prescribe conduct and set standards in some way. While the behaviour that the law prescribes is binding, that prescribed by other sources is not backed by legal force, but garners normative force from professional, clinical, or other authority.

This study highlights the breadth of regulatory sources, all purporting to guide behaviour in different ways, that individuals and institutions who provide assisted dying must be aware of, reconcile, and comply with. These directions might align in terms of the desired behaviour, or they might conflict with one another.

A further key finding is that sources have been developed over time which scaffold the integration of the legal framework into clinical practice.

### Interpretation of Main Findings

This study provides the first holistic understanding of how assisted dying is regulated in Belgium. In doing so, these findings demonstrate that this regulation is multifaceted and complex. Regulation is not something done solely by the State, for example, through the Belgian Act. Rather, regulation is fragmented, in that it is also initiated and enacted by professional bodies, health care organisations and institutions, community groups, and statutory bodies. Fragmentation is also present on a geographical level; different regions have additional significant regulatory sources. In this way, the Belgian assisted dying system is ‘polycentric’, given the influence that non-state regulatory sources exert in this system (Black, 2008). The nature of Belgian assisted dying regulation may make it difficult for practitioners to clearly identify and discern relevant information and obligations. We note there is no centralised repository or resource that identifies these disparate sources of regulation for practitioners working within this framework.

In many cases, regulatory sources might have emerged to address aspects of assisted dying practice on which the Belgian Act is silent. For example, institutional policies commonly address patient ineligibility, aftercare, and patient and relative involvement in assisted dying decision-making. Similarly, the Belgian Act does not address education about assisted dying, so these initiatives were led by universities and other organisations. These examples and the numerous regulatory sources which have a scaffolding function reflect the lack of a well-planned centralised approach to implementing assisted dying in 2002 and in subsequent years. Unlike in other jurisdictions, such as Australia, the Belgian Act did not enter into force after a planned implementation period designed to establish the relevant regulatory infrastructure (White et al., 2021a). Consequently, those involved in assisted dying likely had to develop their own responses to successfully incorporate this legislation into existing policies and processes. The observed fragmentation may be a direct reflection of this ‘bottom up’ regulation generated in response to an essentially ‘hands off’ governmental approach.

While the non-legal regulatory sources engage in some gap-filling, they also provide directions and guidance on many of the same aspects as the law, and each other. For example, a psychiatrist might face a situation where they are determining whether a person requesting an assisted death meets the condition-specific eligibility criteria. The psychiatrist might be guided on this point by their institutional policy, their organisational policy, the training that they have undertaken, the advice of their professional body, and an advisory document produced by an independent statutory body. While it might sometimes be useful for practitioners to have multiple sources of guidance on a particular issue, where directions conflict, confusion and ineffective or inappropriate decision-making might ensue. Different requirements for access to assisted dying across settings have important implications for equity of access for patients. Differences between regulatory directions might account for differences in assisted dying practice on institutional and regional levels (Cohen et al., 2012; Lemiengre et al., 2008). Future research should examine how providers use regulation in their decision-making about assisted dying, and what sources they rely upon when regulatory sources conflict with one another.

The findings in this study are consistent with previous studies investigating the Belgian system which recognise that law, policy, and training programs each influence assisted dying practice (Lemiengre et al., 2010; Verhofstadt et al., 2022; Vissers et al., 2022). Findings are also consistent with research on the role of end-of-life laws; these laws do not create and regulate holistic end-of-life systems on their own (White et al., 2021b).

These findings have important implications for policy makers who develop assisted dying laws and wider regulatory frameworks. For Belgian policy makers, they might highlight the multifaceted nature of this assisted dying regulatory landscape, demonstrate that regulatory sources interact with one another, and show that they each exert differing levels of influence on practice. At an international level, policy makers designing assisted dying frameworks are encouraged to take a holistic approach to see the full breadth of sources of regulation that are likely to guide behaviour, and consider the potential for unintended consequences of the system on patients and providers (Vincent et al., 2020; White et al., 2022).

This study also facilitates inter-jurisdictional comparisons. It does this by shedding new light on the various sources of regulation which might be present in assisted dying systems in different jurisdictions, and the specific functions that each source might have. For example, a recent policy analysis of the Victorian voluntary assisted dying regulatory landscape demonstrates that public policy is far more prominent than regulations developed by professional organisations and health care institutions (Close et al., 2021). In the Dutch system, prosecutorial guidelines and codes of practice play seemingly large regulatory roles (Onwuteaka-Philipsen et al., 2019). For Belgium, public policy is largely absent, and ‘bottom-up’ regulation produced by institutions and organisations appears to dominate this regulatory landscape.

### Limitations

Scoping reviews necessarily limit the sources of information that will be reviewed. Relevant records and regulatory sources may have been missed by utilising the selected databases or the applied search strategies. It is also possible that there are sources of regulation that are not discussed in the literature. Despite this, given the broad framing of the search strategy, and the large pool of initial results yielding a large sample, we are confident that nearly all sources have been captured.

A further limitation is that which sources of regulation were identified and analysed depend on the definition of regulation adopted. This means, for example, that sources beyond law were included but that the impact of individuals in guiding behaviour was excluded. However, while there is debate about what constitutes the concept of regulation, this study did use a widely accepted definition, namely that proposed by Black (2002).

## Conclusion

In this review, we identified several regulatory sources which shape assisted dying practice in Belgium. The Belgian Act is not the sole source of regulation; numerous professional, institutional, organisational, and community-based entities produce sources which seek to shape assisted dying practice. Some of these sources are already well-studied, but the identity and functions of others were largely unknown prior to this study. This study contributes to a more complete understanding of assisted dying regulation in Belgium and facilitates an understanding of the mechanisms regulating organisations use to exert social control over this practice. As assisted dying is increasingly legalised internationally, it is important that policy makers are cognisant of the practical implications of unintended consequences of regulation on providers. Future research should consider the extent to which assisted dying providers are aware of, and impacted by regulation, and which characteristics of regulatory sources determine their impact in practice. Interactions between regulatory sources, for example, inconsistencies, should also be investigated.

## Supplemental Material

Supplemental Material - Mapping Sources of Assisted Dying Regulation in Belgium: A Scoping Review of the Literature Supplemental Material for Mapping Sources of Assisted Dying Regulation in Belgium: A Scoping Review of the Literature by Madeleine Archer, Lindy Willmott, Kenneth Chambaere, Luc Deliens, and Ben P White in OMEGA - Journal of Death and Dying

Supplemental Material - Mapping Sources of Assisted Dying Regulation in Belgium: A Scoping Review of the Literature Supplemental Material for Mapping Sources of Assisted Dying Regulation in Belgium: A Scoping Review of the Literature by Madeleine Archer, Lindy Willmott, Kenneth Chambaere, Luc Deliens, and Ben P White in OMEGA - Journal of Death and Dying

Supplemental Material - Mapping Sources of Assisted Dying Regulation in Belgium: A Scoping Review of the Literature Supplemental Material for Mapping Sources of Assisted Dying Regulation in Belgium: A Scoping Review of the Literature by Madeleine Archer, Lindy Willmott, Kenneth Chambaere, Luc Deliens, and Ben P White in OMEGA - Journal of Death and Dying

Supplemental Material - Mapping Sources of Assisted Dying Regulation in Belgium: A Scoping Review of the Literature Supplemental Material for Mapping Sources of Assisted Dying Regulation in Belgium: A Scoping Review of the Literature by Madeleine Archer, Lindy Willmott, Kenneth Chambaere, Luc Deliens, and Ben P White in OMEGA - Journal of Death and Dying

## References

[bibr1-00302228231210146] Anderson S. Allen P. Peckham S. Goodwin N. (2008). Asking the right questions: Scoping studies in the commissioning of research on the organisation and delivery of health services. Health Research Policy and Systems, 6(1), 7. 10.1186/1478-4505-6-7 18613961 PMC2500008

[bibr2-00302228231210146] Andrew E. V. W. Cohen J. Evans N. Meñaca A. Harding R. Higginson I. Pool R. Gysels M. (2013). Social-cultural factors in end-of-life care in Belgium: A scoping of the research literature. Palliative Medicine, 27(2), 131–143. 10.1177/0269216311429619 22143040

[bibr3-00302228231210146] Arksey H. O’Malley L. (2005). Scoping studies: Towards a methodological framework. International Journal of Social Research Methodology, 8(1), 19–32. 10.1080/1364557032000119616

[bibr4-00302228231210146] Behaegel J. Vercoutere S. Matthys D. (2015). Euthanasia in psychiatric patients. Tijdschrift voor Geneeskunde, 71(17), 1086–1089.

[bibr5-00302228231210146] Belgian Advisory Committee on Bioethics (2019). About Us. Federal public service: Health, food chain safety and environment. https://www.health.belgium.be/en/about-us-1

[bibr6-00302228231210146] Black J. (2002). Critical reflections on regulation. Australian Journal of Legal Philosophy, 27(1), 1.

[bibr7-00302228231210146] Black J. (2008). Constructing and contesting legitimacy and accountability in polycentric regulatory regimes. Regulation & Governance, 2(2), 137.

[bibr8-00302228231210146] Braithwaite J. Coglianese C. Levi-Faur D. (2007). Can regulation and governance make a difference. Regulation & Governance, 1(1), 1–7.

[bibr9-00302228231210146] Close E. Willmott L. White B. P. (2021). Regulating voluntary assisted dying practice: A policy analysis from victoria, Australia. Health Policy, 125(11), 1455–1474. 10.1016/j.healthpol.2021.09.003 34588128

[bibr10-00302228231210146] Cohen J. Chambaere K. (2022). Increased legalisation of medical assistance in dying: Relationship to palliative care. BMJ Supportive & Palliative Care. Advanced Online ahead of print. 10.1136/bmjspcare-2022-003573 35428654

[bibr11-00302228231210146] Cohen J. Van Wesemael Y. Smets T. Bilsen J. Deliens L. (2012). Cultural differences affecting euthanasia practice in Belgium: One law but different attitudes and practices in Flanders and Wallonia. Social Science & Medicine, 75(5), 845–853. 10.1016/j.socscimed.2012.04.021 22682367

[bibr12-00302228231210146] Cohen J. Van Wesemael Y. Smets T. Bilsen J. Onwuteaka-Philipsen B. Distelmans W. Deliens L. (2014). Nationwide survey to evaluate the decision-making process in euthanasia requests in Belgium: Do specifically trained 2nd physicians improve quality of consultation? BMC Health Services Research, 14(2), 307. 10.1186/1472-6963-14-307 25030375 PMC4114442

[bibr13-00302228231210146] Commission fédérale de Contrôle et d’Évaluation de l’Euthanasie (2023). Chiffres de l’année 2022. https://organesdeconcertation.sante.belgique.be/fr/documents/euthanasie-chiffres-de-lannee-2022

[bibr14-00302228231210146] Delbeke E. (2012). Juridische aspecten van zorgverlening aan het levenseinde. Intersentia.

[bibr15-00302228231210146] Gastmans C. Lemiengre J. de Casterlé B. D. (2006). Development and communication of written ethics policies on euthanasia in Catholic hospitals and nursing homes in Belgium (Flanders). Patient Education and Counseling, 63(1–2), 188–195. https://doi.10.1016/j.pec.2005.10.004 16406462 10.1016/j.pec.2005.10.004

[bibr16-00302228231210146] King N. (2004). Using templates in the thematic analysis of text. In Cassell C. Symon G. (Eds), Essential guide to Qualitative methods in Organizational research (pp. 256–270). Sage Publications Ltd. 10.4135/9781446280119.n21

[bibr17-00302228231210146] Lemiengre J. Dierckx de Casterlé B. Denier Y. Schotsmans P. Gastmans C. (2008). How do hospitals deal with euthanasia requests in flanders (Belgium)? A content analysis of policy documents. Patient Education and Counseling, 71(2), 293–301. 10.1016/j.pec.2007.12.010 18296014

[bibr18-00302228231210146] Lemiengre J. Dierckx de Casterlé B. Verbeke G. Guisson C. Schotsmans P. Gastmans C. (2007). Ethics policies on euthanasia in hospitals—a survey in Flanders (Belgium). Health Policy, 84(2–3), 170–180. 10.1016/j.healthpol.2007.05.007 17618011

[bibr19-00302228231210146] Lemiengre J. Gastmans C. Schotsmans P. Dierckx de Casterlé B. (2010). Impact of written ethics policy on euthanasia from the perspective of physicians and nurses: A multiple case study in hospitals. AJOB Primary Research, 1(2), 49–60. 10.1080/21507716.2010.489347

[bibr20-00302228231210146] Lewis P. Black I. (2013). Reporting and scrutiny of reported cases in four jurisdictions where assisted dying is lawful: A review of the evidence in The Netherlands, Belgium, Oregon and Switzerland. Medical Law International, 13(4), 221–239. 10.1111/jlme.12098 24446946

[bibr21-00302228231210146] Onwuteaka-Philipsen B. Willmott L. White B. P. (2019). Regulating voluntary assisted dying in Australia: Some insights from The Netherlands. Medical Journal of Australia, 211(10), 438. 31494938 10.5694/mja2.50310 PMC6900053

[bibr22-00302228231210146] Tricco A. C. Lillie E. Zarin W. O’Brien K. K. Colquhoun H. Levac D. Moher D. Peters M. D. J. Horsley T. Weeks L. Hempel S. Akl E. A. Chang C. McGowan J. Stewart L. Hartling L. Aldcroft A. Wilson M. G. Garritty C. Straus S. E. (2018). PRISMA extension for scoping reviews (PRISMA-ScR): Checklist and explanation. Annals of Internal Medicine, 169(7), 467–473. 10.7326/M18-0850 30178033

[bibr23-00302228231210146] Vansweevelt T. (2003). De euthanasiewet: De ultieme bevestiging van het zelfbeschikkingsrecht of een gecontroleerde keuzevrijheid. Tijdschrift Voor Gezondheidsrecht, 4(22), 63.

[bibr24-00302228231210146] Van Wesemael Y. Cohen J. Onwuteaka-Philipsen B. D. Bilsen J. Distelmans W. Deliens L. (2009). Role and involvement of life end information forum physicians in euthanasia and other end-of-life care decisions in Flanders, Belgium. Health Services Research, 44(6), 2180–2192. 10.1111/j.1475-6773.2009.01042.x 19780854 PMC2796321

[bibr25-00302228231210146] Verhofstadt M. Audenaert K. Van Assche K. Sterckx S. Chambaere K. (2019a). Ghent University Hospital’s protocol regarding the procedure concerning euthanasia and psychological suffering. BMC Medical Ethics, 20(1), 59. 10.1186/s12910-019-0400-z 31477106 PMC6720846

[bibr26-00302228231210146] Verhofstadt M. Chambaere K. Pardon K. Mortier F. Liégeois A. Deliens L. Audenaert K. (2022). The impact of the euthanasia assessment procedure: A qualitative interview study among adults with psychiatric conditions. BMC Psychiatry, 22(1), 435. 10.1186/s12888-022-04039-2 35761195 PMC9235145

[bibr27-00302228231210146] Verhofstadt M. Van Assche K. Sterckx S. Audenaert K. Chambaere K. (2019b). Psychiatric patients requesting euthanasia: Guidelines for sound clinical and ethical decision making. International Journal of Law and Psychiatry, 64(1), 150–161. 10.1016/j.ijlp.2019.04.004 31122625

[bibr28-00302228231210146] Vincent C. Oikonomou E. Carthey J. Macrae C. (2020) Redesigning safety regulation in the NHS, BMJ. 368, m760). 10.1136/bmj.m760 32179534

[bibr29-00302228231210146] Vissers S. Dierickx S. Chambaere K. Deliens L. Mortier F. Cohen J. (2022). Assisted dying request assessments by trained consultants: Changes in practice and quality - repeated cross-sectional surveys (2008–2019). BMJ Supportive & Palliative Care. Advanced Online ahead of print. 10.1136/spcare-2021-003502 35768204

[bibr30-00302228231210146] White B. P. Willmott L. Close E. (2022). Better regulation of end-of-life care: A call for a holistic approach. Journal of bioethical inquiry. Advanced Online ahead of print. 10.1007/s11673-022-10213-8 PMC9908626 36251135

[bibr31-00302228231210146] White B. P. Willmott L. Close E. Hewitt J. Meehan R. Greaves L. L. Parker M. H. Yates P. (2021a). Development of voluntary assisted dying training in victoria, Australia: A model for consideration. Journal of Palliative Care, 36(3), 162–167. 10.1177/0825859720946897 32752924

[bibr32-00302228231210146] White B. P. Willmott L. Feeney R. Neller P. Then S.-N. Bryant J. Waller A. Yates P. (2021b). Limitations in health professionals’ knowledge of end-of-life law: A cross-sectional survey. BMJ Supportive & Palliative Care. Advanced Online ahead of print. 10.1136/bmjspcare-2021-003061 34083318

