# Supplementary material for: Mapping Sources of Assisted Dying Regulation in Belgium: A Scoping Review of the Literature
Source: Omega (Westport). 2023 Nov 1;92(3):1610–33. doi: 10.1177/00302228231210146 (PMC12769923; doi:10.1177/00302228231210146)
Supplement: Supplemental Material - Mapping Sources of Assisted Dying Regulation in Belgium: A Scoping Review of the Literature [file sj-pdf-1-ome-10.1177_00302228231210146.pdf]

Scoping review protocol

Overview of the study

- Euthanasia is regulated in Belgium by a variety of sources
- People and institutions have to make decisions about euthanasia i.e. how to handle a first request, how to navigate a conscientious objection, how to interpret the due care criteria and eligibility requirements, how to handle the outcome of consultation, how to choose an administration substance and administration method, etc. What regulatory sources exist and how do they inform decision-making about euthanasia?
- A regulatory perspective (i.e. examining the mechanics and structure of the system, identification of how decisions are made) is helpful and necessary to identify the working parts of this system and see how they might work together to guide decision-making about euthanasia
- There is much discussion of various regulatory components of the Belgian euthanasia regime in the literature e.g. training, policy, law
- Regulatory literature has been siloed. To understand regulation, need to integrate for holistic picture. This has implications for a better understanding, learnings and improvements.

Objectives of the study

Overarching

- How is euthanasia regulated in Belgium? What are the mechanisms and sources of regulation?
- What are the regulatory influences on how people/institutions make decisions about euthanasia, and what is the nature of this regulatory influence?

Specific

- To identify, describe and summarise what sources of regulation are active in the Belgian euthanasia regime (as identified in the literature)
- To identify, describe, and summarise the regulatory functions of these sources (as identified in the literature)

Research question(s)

What are the sources of regulation operating in the current Belgian euthanasia regime – including, but not limited to law, policy, ethics, training, system architecture and professional standards – and what are the regulatory functions of these sources?

Key concepts

- Regulation
  - 'Regulation is the sustained and focused attempt to alter the behaviour of others according to defined standards or purposes with the intention of producing a broadly identifiable outcome or outcomes, which may involve mechanisms of standard-setting, information-gathering and behaviour-modification' (Julia Black, 'Critical Reflections on Regulation' (2002) 27 Australian Journal of Legal Philosophy 1, 26).
  - This definition captures the multiplicity and plurality of regulatory sources including non-state actors (regulation is not confined to State actors)
  - Conveys intentionality in regulatory sources having to deliberately attempt to impact decision-making about AD
  - Regulation occurs through a variety of sources e.g. law, policy, ethics, training, professional standards, etc
  - Our conception of regulation includes understanding both how the practice is regulated on paper and how regulation works in practice (both are relevant to identify how people make decisions about euthanasia). How the system is implemented has regulatory implications (influences decision-making), as do the support systems that are provided.
- Source of regulation
  - As informed by the adopted definition of regulation.
  - In order to be a source of regulation the person/group/thing/instrument must intend, in a sustained and focused way, to influence the practice of euthanasia in Belgium.
  - Practically, because intention is subjective etc, if a person/group/thing/instrument says/does something to others about how euthanasia should operate = source of regulation.

    - Sources of regulation, following on from definition of ‘regulation’ can be both State or non-State based.
    - e.g. ‘culture’ does not intend, in a sustained and focused way, to influence decision-making about euthanasia.
    - Note: Blanket exclusion of non-Belgian regulation (e.g. European, international) to focus in on specific, operational regulation within Belgium (02/03/22).
    - Note: Exclusion of individual actors (includes individuals, advance directives), consistent with this paper’s definition of regulation given ‘sustained and focused’ requirement (02/03/22) and focus on structures and systems rather than e.g. advocacy efforts (01/10/22).
- Regulatory role/function
    - What actual influence does this source have on decision-making about euthanasia (decision-making about the practice of euthanasia from the first request to aftercare)? What is the nature of this influence?
    - Anticipated regulatory functions of regulatory sources might include:
        - Operationalising legal due care criteria
        - Process-setting
        - Determining participation/access (of patients and practitioners)
        - Definitional and interpretational
        - Oversight and enforcing compliance
- Euthanasia
    - Includes voluntary euthanasia and physician assisted suicide. As defined/legalised by the Belgian Act on Euthanasia (active ending of life at the explicit request of the patient)(excl. involuntary and non-voluntary forms).

Broad scoping review method

To be guided by Arksey and O’Malley’s five-stage scoping review methodological framework (Hilary Arksey and Lisa O’Malley, ‘Scoping Studies: Towards a Methodological Framework’ (2005) 8(1) International Journal of Social Research Methodology 19).

- Stage 1: identify the research question
- Stage 2: identify relevant studies
- Stage 3: selects the studies

- Stage 4: charts the data
- Stage 5: collates, summarises and reports the results

Review inclusion criteria

- Journal article in a peer reviewed journal (or book chapter (25/03/22)).
- Full text available/able to be obtained, (if no abstract, take inclusive approach and proceed to full-text review).
- 28 May 2002 (inclusive) to present (to align with the research question capturing *current* sources of regulation, 2002 was the year the law was passed, not looking at euthanasia prior to the law, only regulation since the law).
- In English (search expanded to Dutch and French search terms on 09/03/22).
- Belgian regulation of euthanasia is a substantive focus of the paper

Review search strategy

- Databases scoped: EBSCO host (searching Psych Info, Legal Source, Cinahl, Medline), Scopus, Pubmed.
    - Rationale for inclusion/exclusion of databases. Few relevant articles/already captured in other databases: Web of Science, Embase, Westlaw International, Ageline, Social Work Abstracts. Inability to make consistent search terms in Google Scholar and HeinOnline (vast majority of articles already captured in HeinOnline.
    - Each of the included databases included some unique articles not already captured. Cover law and health papers.
- Search strategy is to target 2 criteria and a number of variations thereupon: (1) euthanasia and (2) Belgium within the title or the abstract of the paper
    - Euthanasia: Euthanasia; Voluntary euthanasia; Assisted dying; Assisted suicide; Assisted death; Physician assisted suicide; Physician assisted death; Physician assisted dying; Mercy killing; Medical assistance in dying; Medical aid in dying
    - Belgium: Belg* (covers Belgian, Belgium); Flemish; Flanders; Walloon; Wallonia; Benelux
- Limiters/filters in databases
    - Type of document: journal

- 2002-present
- Peer reviewed (if the option presents)
- Language – English, French, Dutch

- Notes:
  - Searching periods will be limited.
  - These methods will be developed over the course of the study.
- Supplement the review sample with reference list searching (from already included records)

Specific scoping review method

This part of the method commences once all databases have produced a list of papers containing the results of the search strategy. Zotero will be used to collate and remove duplicates.

- First: pilot this protocol with 20 abstracts
- Remove duplicates.
- <u>Title screening.</u> Inclusion criteria applied to title. Inclusive approach will be applied due to limited information contained in title. Remove clearly irrelevant papers e.g. relate to euthanasia of animals (N = X) AND papers which have slipped through but which do not meet inclusion criteria e.g. are in another language, not the journal format specified in the inclusion criteria, etc.
- <u>Abstract screening.</u> Inclusion criteria applied to abstract. This is to be a robust screening stage but still take an inclusive approach. In order to satisfy 'substantive discussion of regulatory source', abstract must demonstrate this (show that a regulatory source will be substantively discussed/analysed or the paper is about analysing a regulatory source (N = X). Not constrained in this process by where in the abstract the inclusion criteria are met i.e. whether it is the objective of a paper or in its findings. An inclusive approach will be applied in this phase.
- Two authors to co-assess 30 abstracts and then lead author to complete the sample.
- Moderation to be completed blind to each moderator's thoughts, and disagreements to be resolved by discussing and reaching a view.
- <u>Full-text screening</u>. Inclusion criteria applied to full-text paper. Where a full-text reviewed paper meets the inclusion criteria, its references will be scanned and any

relevant papers it produces will be subject to the above processes and may be included (N = X).

- Two authors to moderate a sample of 10 records using the same moderation processes as above.
- Final sample included in the review (N = X)

Data extraction

Revised during screening and full-text review.

Lead author first to familiarise with all articles by reading in full (without any data extraction)

Extracted data to include (unless adapted):

- Paper title/reference
- Type of study/paper (e.g. empirical)
- Focus of article (descriptive, e.g. hospital policies on euthanasia in Flanders)
- Source(s) of regulation (e.g. Act on euthanasia, Flemish nursing home policies on euthanasia or reported on a more macro level)
- Who is responsible for/makes the source of regulation (e.g. Ministry of Justice, Caritas Catholica)
    - What does the paper say about the regulatory source (non-critical, descriptive)?
    - What functions of the source of regulation does the paper describe? (e.g. interpretation, permissiveness of an act, approach, method)
    - What issues are subject to regulation in the regulatory sources discussed in the literature (e.g. institutional objection, eligibility for minors).
- Notes/regulatory observations

Data analysis

- Processes of extracting data about and analysing data on regulatory sources overlap. This is because a coding frame is used to hierarchically map and organise the data, guided by *a priori* conceptions of regulatory sources from existing scholarship.

- Thematic analysis of regulatory function data. Codebook approach to thematic analysis using *a priori* themes imposed deductively.

Results

- Descriptive statistics
- Part 1 – factual exploration of the regulatory sources which exist in this regulatory system (the mapping analysis).
- Part 2 – presentation on themes as to regulatory functions
