# Supplementary material for: Mapping Sources of Assisted Dying Regulation in Belgium: A Scoping Review of the Literature
Source: Omega (Westport). 2023 Nov 1;92(3):1610–33. doi: 10.1177/00302228231210146 (PMC12769923; doi:10.1177/00302228231210146)
Supplement: Supplemental Material - Mapping Sources of Assisted Dying Regulation in Belgium: A Scoping Review of the Literature [file sj-pdf-2-ome-10.1177_00302228231210146.pdf]

Search strategy information

Three separate searches in the databases were undertaken

(1) Initial database search to capture only English journal articles
(2) Expansion of initial search to capture Dutch and French journal articles
(3) Expansion of initial search to capture book chapters in English

Initial Database Search (search undertaken 11/02/22)

Database	Search Strategy	Limiters
EBSCOhost (Legal Source, APAPsychInfo, Medline, Cinahl)	S1: AB (euthanasia OR "voluntary euthanasia" OR "assisted dying" OR "assisted suicide" OR "assisted death" OR "physician assisted suicide" OR "physician assisted death" OR "physician assisted dying" OR "mercy killing" OR "medical assistance in dying" OR "medical aid in dying") AND AB (belg* OR flemish OR flanders OR walloon OR wallonia OR benelux)<br><br>S2: TI (euthanasia OR "voluntary euthanasia" OR "assisted dying" OR "assisted suicide" OR "assisted death" OR "physician assisted suicide" OR "physician assisted death" OR "physician assisted dying" OR "mercy killing" OR "medical assistance in dying" OR "medical aid in dying") AND TI (belg* OR flemish OR flanders OR walloon OR wallonia OR benelux)<br><br>S1 OR S2	From 2002-2022<br><br>Peer reviewed<br><br>Academic journals<br><br>Dutch; Flemish; English; French
Scopus	(TITLE ((euthanasia OR "voluntary euthanasia" OR "assisted dying" OR "assisted suicide" OR "assisted death" OR "physician assisted suicide" OR "physician assisted death" OR "physician assisted dying" OR "mercy killing" OR "medical assistance in dying" OR "medical aid in dying") AND (belg* OR flemish OR flanders OR walloon OR wallonia OR benelux))) OR (ABS ((euthanasia OR "voluntary euthanasia" OR "assisted dying" OR "assisted suicide" OR "assisted death" OR "physician assisted suicide" OR "physician assisted death" OR "physician assisted dying" OR "mercy killing" OR "medical assistance in dying" OR "medical aid in dying") AND (belg* OR flemish OR flanders OR walloon OR wallonia OR benelux)))	2002-2022<br><br>Article<br><br>Source type: Journal<br>English, French, Dutch (exclude Spanish)
Pubmed	(euthanasia[Title/Abstract] OR "voluntary euthanasia"[Title/Abstract] OR "assisted dying"[Title/Abstract] OR "assisted suicide"[Title/Abstract] OR "assisted death"[Title/Abstract] OR "physician assisted suicide"[Title/Abstract] OR "physician assisted death"[Title/Abstract] OR "physician assisted	2002-2022<br><br>Dutch, English,

	dying"[Title/Abstract] OR "mercy killing"[Title/Abstract] OR "medical assistance in dying"[Title/Abstract] OR "medical aid in dying"[Title/Abstract]) AND (belg*[Title/Abstract] OR flemish[Title/Abstract] OR flanders[Title/Abstract] OR walloon[Title/Abstract] OR wallonia[Title/Abstract] OR benelux[Title/Abstract])	French

Second Database Search to specifically capture French and Dutch journal articles (search undertaken 09/03/22)

Database	Search Strategy	Limiters
EBSCOhost (Legal Source, APAPsychInfo, Medline, Cinahl)	French title search (and same for abstract): euthanasie OR "aide medicale a mourir" OR "aide au suicide" OR "aider a mourir" AND belg* OR flamand OR flandre OR wallonie OR Benelux<br><br>Dutch title search (and same for abstract): euthanasie AND belg* OR vlaam OR vlaanderen OR waals OR wallonie OR benelux	
Scopus	French<br>(TITLE ((euthanasie OR "aide medicale a mourir" OR "aide au suicide" OR "aider a mourir") AND (belg* OR flamand OR flandre OR wallonie OR benelux))) OR (ABS ((euthanasie OR "aide medicale a mourir" OR "aide au suicide" OR "aider a mourir") AND (belg* OR flamand OR flandre OR wallonie OR benelux)))<br><br>Dutch<br>(TITLE ((euthanasie) AND (belg* OR vlaam OR vlaanderen OR waals OR wallonie OR benelux))) OR (ABS ((euthanasie) AND (belg AND *or AND vlaam OR vlaanderen OR waals OR wallonie OR benelux)))	2002-2022, Journal article, French<br><br>2002-2022, Dutch, Source type: journal
Pubmed	French<br>(euthanasie[Title/Abstract] OR "aide medicale a mourir"[Title/Abstract] OR "aide au suicide"[Title/Abstract] OR "aider a mourir"[Title/Abstract]) AND (belg*[Title/Abstract] OR flamand[Title/Abstract] OR flandre[Title/Abstract] OR wallonie[Title/Abstract] OR benelux[Title/Abstract])<br><br>Dutch<br>(euthanasie[Title/Abstract]) AND (belg*[Title/Abstract] OR vlaam[Title/Abstract] OR	2002-2022, French<br><br>2002-2022,

	vlaanderen[Title/Abstract] OR waals[Title/Abstract] OR wallonie[Title/Abstract] OR benelux[Title/Abstract])	Dutch

Third database search to capture book chapters in English (undertaken on 25/03/22)

**Database**	**Search Strategy**	**Limiters**
EBSCOhost (Legal Source, APAPsychInfo, Medline, Cinahl)	S1 or S2<br><br>S1 = AB (euthanasia OR "voluntary euthanasia" OR "assisted dying" OR "assisted suicide" OR "assisted death" OR "physician assisted suicide" OR "physician assisted death" OR "physician assisted dying" OR "mercy killing" OR "medical assistance in dying" OR "medical aid in dying") AND AB (belg* OR flemish OR flanders OR walloon OR wallonia OR benelux)<br><br>S2 = TI (euthanasia OR "voluntary euthanasia" OR "assisted dying" OR "assisted suicide" OR "assisted death" OR "physician assisted suicide" OR "physician assisted death" OR "physician assisted dying" OR "mercy killing" OR "medical assistance in dying" OR "medical aid in dying") AND TI (belg* OR flemish OR flanders OR walloon OR wallonia OR benelux)	2002-2022<br><br>Book
Scopus	(TITLE ((euthanasia OR "voluntary euthanasia" OR "assisted dying" OR "assisted suicide" OR "assisted death" OR "physician assisted suicide" OR "physician assisted death" OR "physician assisted dying" OR "mercy killing" OR "medical assistance in dying" OR "medical aid in dying") AND (belg* OR flemish OR flanders OR walloon OR wallonia OR benelux))) OR (ABS ((euthanasia OR "voluntary euthanasia" OR "assisted dying" OR "assisted suicide" OR "assisted death" OR "physician assisted suicide" OR "physician assisted death" OR "physician assisted dying" OR "mercy killing" OR "medical assistance in dying" OR "medical aid in dying") AND (belg* OR flemish OR flanders OR walloon OR wallonia OR benelux)))	2002-2022<br><br>Source type: book<br><br>Book chapter
Pubmed	(euthanasia[Title/Abstract] OR "voluntary euthanasia"[Title/Abstract] OR "assisted dying"[Title/Abstract] OR "assisted suicide"[Title/Abstract] OR "assisted death"[Title/Abstract] OR "physician assisted suicide"[Title/Abstract] OR "physician assisted death"[Title/Abstract] OR "physician assisted dying"[Title/Abstract] OR "mercy killing"[Title/Abstract] OR "medical assistance in dying"[Title/Abstract] OR	2002-2022<br><br>Books and documents

	"medical aid in dying"[Title/Abstract]) AND (belg*[Title/Abstract] OR flemish[Title/Abstract] OR flanders[Title/Abstract] OR walloon[Title/Abstract] OR wallonia[Title/Abstract] OR benelux[Title/Abstract])	
