# Supplementary material for: Mapping Sources of Assisted Dying Regulation in Belgium: A Scoping Review of the Literature
Source: Omega (Westport). 2023 Nov 1;92(3):1610–33. doi: 10.1177/00302228231210146 (PMC12769923; doi:10.1177/00302228231210146)
Supplement: Supplemental Material - Mapping Sources of Assisted Dying Regulation in Belgium: A Scoping Review of the Literature [file sj-pdf-4-ome-10.1177_00302228231210146.pdf]

Regulatory sources scoped in the review sample

**Regulatory Source**	**Sub-category**	**Regulatory Instrument**	**Provenance**
**Law**	Statute	Belgian Act on Euthanasia 2002 - *Loi relative à l'euthanasie/ Wet betreffende de euthanasie*	Legislature (Ministry of Justice)
		Amendment – 10 November 2005 - *Act supplementing the Act of 28 May 2002 on euthanasia with provisions concerning the role of the pharmacist and the use and availability of euthanising substances*	
		Amendment – 28 February 2014 - *Act amending the Act of 28 May 2002 on euthanasia, with a view to extending euthanasia to minors*	
		Amendment – 15 March 2020 – *Law amending the legislation on euthanasia*	
	Royal decrees	Royal Decree of 3 February 2003 - *Establishing the framework for the administrative staff of the Federal Control and Evaluation Commission set up for the application of the law on euthanasia*	The Crown
		Royal Decree of 2 April 2003 - *Laying down the procedures for drawing up, reconfirming, revising or withdrawing the advance declaration on euthanasia*	
		Royal Decree of 27 April 2007 - *Regulating the manner in which the advance declaration of euthanasia is registered and communicated via the National Register to the doctors concerned*	
	Case law – constitutional law	Constitutional court judgement 14 January 2004 – Judgement number 4/2004	Constitutional court
		Constitutional court judgement of 29 October 2015 – Judgement number 153/2015	Constitutional court
	Case law – Criminal law	Judgement of 31 January 2020, criminal trial and acquittal of three physicians	East Flanders Court sitting in Ghent/Ghent Assizes
		Judgement of 6 February 2003, decision not to prosecute two physicians	Criminal Court of Liège
		Judgement of 9 December 2004 KI Ghent 9 December 2004, T Gez 2007-2008, 39	Ghent Court of Appeal
**Policy**	Organisation-level policies (Healthcare institution	Caring for a Dignified End of Life (Zorg voor een menswaardig levenseinde) published in 2005	Caritas Flanders
		Facing requests for euthanasia (clinical practice guideline) published 2004	

	umbrella organisations, healthcare networks)	Ethisch advies 20: Levenseindezorg voor niet-terminale patienten met ernstige psychiatrische aandoeningen van 23 Januari 2018 - *End of Life Care for non-terminally ill patients with serious psychiatric disorders*	Zorgnet-Icuro
		Omgaan met euthanasie en andere vormen van medisch begeleid sterven [Dealing with euthanasia and other forms of medically assisted dying], published 6 September 2003	Palliative Care Flanders (formerly the Flemish Federation for Palliative Care)
		Treatment decisions in advanced disease – A conceptual framework (15 September 2006)	
		Over palliatieve zorg en euthanasie [On palliative care and euthanasia], reference document (2011)	
		End-of-life care: No twin-track policy (2008)	
		Euthanasie: texte de consensus des trois fédérations de soins Palliatifs 2013 – *Euthanasia: consensus text of the three palliative care federations*	Belgian palliative care federations (Federation for Palliative Care, Fédération Wallonne des Soins Palliatifs and the Fédération Pluraliste Bruxelloise de Soins Palliatifs et Continus)
		Euthanasia: The point of view of the Congregation of the Brothers of Charity. R Stockman, 2002. Published in Ghent, Belgium: Brothers of Charity Publications.	Brothers of Charity
		Advice on the counselling of psychiatric patients with a request for euthanasia (2006)	
		Opinion on euthanasia on grounds of psychological suffering in non-dying conditions 2017. Vision of Brothers of Charity of Belgium on euthanasia for psychological suffering in non-terminal situations (March 2017)	
	Institution-level policies (Healthcare institutions)	Ethics policies on euthanasia (generally)	Flemish hospitals, Flemish nursing homes, Flemish catholic nursing homes, Flemish health care institutions (nursing homes and hospitals), institutions, intramural health care settings in Flanders (general hospitals, psychiatric hospitals, institutions for people with a mental disability)
		Procedure concerning euthanasia and psychological suffering, 2009	Ghent University Hospital

		Protocol for assessments of euthanasia requests for psychological suffering	University Hospital Brussels
		Protocol for assessment of euthanasia requests for psychological suffering	University Hospital Louvain/Leuven
	Public policy	Circular addressed to doctors, “Advance requests for euthanasia – electronic consultation by doctors” 4 September 2008[i]	Federal Health Department
**Professional standards**	Written standards – Physicians (in general)	Advice of March 2003 regarding palliative care, euthanasia, and other medical decisions at the end of life	Belgian National Council of Physicians/Order of Physicians (National)
		Code of Medical Deontology/Code of Ethics	
		Deontological Code to refer to patients in case of refused euthanasia – Advice from the Belgian National Board 2017	
		Deontological Code on the Management of Psychiatric Euthanasia Requests 2019	
		Advice of 18 or 21 January 2014 concerning the extension to minors regarding the 2002 Act on Euthanasia (2014a)	Royal Belgian Academy of Medicine/Académie Royale de Belgique (Wallonia)
		Advice about the law on euthanasia extended to children (2014b)	
	Written standards – Psychiatrists	How to deal with euthanasia requests from psychiatric patients within the legal framework – December 2017	Flemish Association for Psychiatry (Flanders)
		Reporting form regarding consultation on requests for AD from psychiatric patients	
		Report on euthanasia for psychological suffering	Royal Society of Psychiatric Medicine of Belgium (Société Royale de Médecine Psychiatrique de Belgique)
	Written standards – General practitioners	Position on Medical End of Life Decisions and Euthanasia 2003	Scientific Society of Flemish General Practitioners
	Written standards – Pharmacists	Guidelines for pharmacists on the euthanasia legislation	Association Pharmaceutique Belge (APB)(Belgian Pharmaceutical Association)
	Disciplinary proceedings	Decision 24 October 2007	Provincial Council of West Flanders (Order of Physicians)
**Training**	Mandatory tertiary education	Undergraduate core curriculum	Vrije Universiteit Brussel
	Non-mandatory further tertiary education	Curriculum of Master’s degree certificate "Pain and the End of Life"	Université Libre de Bruxelles
		Post-graduate interuniversity course on palliative care	Flemish Federation for

			Palliative Care
	Non-mandatory community-initiated regional training programs	LEIF training program/curriculum	Life End Information Forum (services Brussels and Flanders)
		Forum End of Life training program/curriculum	Forum End of Life (services Wallonia)
**Advisory documents**	Independent statutory bodies (independent bodies created pursuant to statute and who report to Parliament)	CFCEE information brochure	Federal Control and Evaluation Commission (CFCEE)
		CFCEE biannual reports	
		Opinion on the ethical aspects of the application of the 2002 law dealing with the conscience clause – Opinion no 59 - 27 January 2014	Belgian Advisory Committee on Bioethics (BACB) (Comité consultatif de Bioéthique de Belgique)
		Opinion on euthanasia in case of non-terminally ill patients, psychological suffering and psychiatric disorders – opinion no 73 – 11 September 2017	
	Academia[b]	'L'euthanasie: Considérations <practiques>' [2006] 230 *La Revue de la Médecine Générale* 82	Dr Beatrice Figa, general practitioner, Brussels
**System design**	Pre-existing system design		Public prosecutor
	Created pursuant to the euthanasia legislation	CFCEE registration document	CFCEE
			CFCEE secretariat
		National database/register of Advance Directives on Euthanasia	Federal Health Department
	Developed independently of the Belgian Act	Consultation centres	Life End Information Forum
			Forum End of Life
			ULTeam (Team Uitklaring Levensein vragen) established 2011
			Other end-of-life consultation services: Jules-Borden Institute, Brugmann Hospital in Brussels, Citadelle Hospital Liege, ULTeam, LEIF West-Flanders (2013), LEIF Ghent (2015)

[a] This source was not able to be accessed due to a broken link on the Department of Health's website: https://www.health.belgium.be/fr/sante/prenez-soin-de-vous/debut-et-fin-de-vie/euthanasie/euthanasie-medecins#DeclarationAnticipee but it was included as a regulatory source as it was indicated in text to meet the study's definition of regulation.

[b] This source was included as a regulatory source because the record discussed that it was widely disseminated to general practitioners as a form of training.
